# High similarity in the microbiota of cold-water sponges of the Genus *Mycale* from two different geographical areas

**DOI:** 10.7717/peerj.4935

**Published:** 2018-06-07

**Authors:** César A. Cárdenas, Marcelo González-Aravena, Alejandro Font, Jon T. Hestetun, Eduardo Hajdu, Nicole Trefault, Maja Malmberg, Erik Bongcam-Rudloff

**Affiliations:** 1 Departamento Científico, Instituto Antártico Chileno, Punta Arenas, Chile; 2 Marine Biodiversity Group, Department of Biology, University of Bergen, Bergen, Norway; 3 Museu Nacional, Departamento de Invertebrados, Universidade Federal do Rio de Janeiro, Rio de Janeiro, Brazil; 4 GEMA Center for Genomics, Ecology & Environment, Universidad Mayor, Santiago, Chile; 5 SLU Global Bioinformatics Centre, Department of Animal Breeding and Genetics, Swedish University of Agricultural Sciences, Uppsala, Sweden; 6 Section of Virology, Department of Biomedical Sciences and Veterinary Public Health, Swedish University of Agricultural Sciences, Uppsala, Sweden

**Keywords:** Porifera, 16S rRNA, High throughput sequencing, Antarctica, Subantarctic, Magallanes, Cold-water filter feeders

## Abstract

Sponges belonging to genus *Mycale* are common and widely distributed across the oceans and represent a significant component of benthic communities in term of their biomass, which in many species is largely composed by bacteria. However, the microbial communities associated with *Mycale* species inhabiting different geographical areas have not been previously compared. Here, we provide the first detailed description of the microbiota of two *Mycale* species inhabiting the sub-Antarctic Magellan region (53°S) and the Western Antarctic Peninsula (62–64°S), two geographically distant areas (>1,300 km) with contrasting environmental conditions. The sponges *Mycale (Aegogropila) magellanica* and *Mycale (Oxymycale) acerata* are both abundant members of benthic communities in the Magellan region and in Antarctica, respectively. High throughput sequencing revealed a remarkable similarity in the microbiota of both sponge species, dominated by *Proteobacteria* and *Bacteroidetes*, with both species sharing more than 74% of the OTUs. In contrast, 16% and 10% of the OTUs were found only in either *M. magellanica* or *M. acerata*, respectively. Interestingly, despite slight differences in the relative abundance, the most dominant OTUs were present in both species, whereas the unique OTUs had very low abundances (less than 1% of the total abundance). These results show a significant overlap among the microbiota of both *Mycale* species and also suggest the existence of a low level of specificity of the most dominant symbiont groups.

## Introduction

Sponges can host diverse and abundant microbial communities (Hentschel et al., 2012; Taylor et al., 2007; Thacker & Freeman, 2012; Thomas et al., 2016; Webster & Taylor, 2012), and are considered reservoirs of exceptional microbial diversity and a major contributor to the total microbial diversity of the world’s oceans (Thomas et al., 2016). In some cases, symbionts can comprise large parts of the sponge volume (Hentschel et al., 2003; Taylor et al., 2007; Ribes et al., 2012). Sponge hosts can benefit from microbial symbionts as the latter can provide supplemental nutrition (Moitinho-Silva et al., 2017; Sarà et al., 1998) and secondary metabolites (Flatt et al., 2005; Schmidt, 2008). It has been suggested that microbes can also enhance structural rigidity (Wilkinson et al., 1981), provide protection from UV radiation (Regoli et al., 2000; Usher, 2008) and protection from, or deterrence of, predators (Pawlik et al., 1995). Recent literature has demonstrated that microbial symbionts are important in carbon cycling, by removing important amounts of carbon (De Goeij et al., 2013); however, in many cases their exact role is still a matter of debate (See Hoer et al., 2018; Maldonado, Ribes & Van Duyl, 2012; McMurray et al., 2018).

Previous studies have addressed the degree of host specificity in closely related sponge species and at different temporal and spatial scales (Erwin, Olson & Thacker, 2012; Erwin et al., 2012a; Erwin et al., 2012b; Marino et al., 2017; Montalvo & Hill, 2011; Reveillaud et al., 2014; Souza et al., 2017). These studies have provided important insights on the evolutionary and ecological factors driving relationships between microbes and their hosts. However, only recently some research has assessed the impact of host phylogeny and identity on microbial communities (see Easson & Thacker, 2014; Thomas et al., 2016). Results from these studies suggest a significant role of host phylogeny, however specific members of the microbiota of each host are also strongly influenced by specific factors such as habitat, nutrients, external and internal structure of the host. Furthermore, some studies have addressed the degree of specificity in high microbial abundance (HMA) and low microbial abundance (LMA) sponges, showing a high degree of specificity for both categories (De Mares et al., 2017; Easson & Thacker, 2014; Erwin et al., 2015); however, according to some studies, LMA sponges tend to host less specific microbial communities which also tend to be more similar to the communities in the surrounding water (e.g., Björk et al., 2013; Giles et al., 2013; Erwin et al., 2015).

In addition, a number of studies have assessed the stability of microbial communities when exposed to different stressors, reporting variable results (see Pita et al., 2018 for a review). While several studies supported the high stability of sponge-associated microbial communities under different environmental conditions (Björk et al., 2013; Cárdenas et al., 2014; Erwin et al., 2012b; Erwin et al., 2015; Simister et al., 2013), others have reported shifts in the sponge-associated microbiota under thermal stress (Lemoine et al., 2007; Webster, Cobb & Negri, 2008; Fan et al., 2013) or when exposed to combined factors such as sedimentation and light availability (Pineda et al., 2016; Pineda et al., 2017), suggesting it can be a good indicator of environmental variation. This is especially relevant in the context of climate change, considering the Western Antarctic Peninsula (WAP) is one of the areas that is experiencing one of the fastest rates of warming on Earth (Cook et al., 2016; Meredith & King, 2005).

Despite the importance of sponges in Antarctic environments in terms of their abundance and the role they play as habitat providers and important source of nutrients for several species (Cárdenas et al., 2016; Dayton et al., 1974; McClintock et al., 2005), research on sponge-microbe associations in Antarctica is still very scarce, and only a few sponges species have been studied (Rodríguez-Marconi et al., 2015; Webster et al., 2004). The studies available have demonstrated that Antarctic sponges host a wide range of microorganisms, including bacteria, archaea, diatoms and dinoflagellates (Cerrano et al., 2000; Webster et al., 2004). Webster et al. (2004) using clone libraries and denaturing gradient gel electrophoresis (DGGE) reported consistent microbial communities from five sponge species collected across three sites in McMurdo Sound, Ross Sea (East Antarctica). The authors described diverse bacterial communities with a significant proportion of them being sponge-specific (40% of unique banding patterns), dominated by *Proteobacteria* and *Bacteroidetes*. A more recent study, characterizing the microbial communities of single specimens from eight different sponges from King George Island, South Shetland Islands (Maritime Antarctica) using high throughput 16S and 18S sequencing, reported highly diverse bacterial communities also dominated by *Proteobacteria* and *Bacteroidetes* (Rodríguez-Marconi et al., 2015), suggesting they might correspond to LMA sponges. However, studies on bacterial communities associated with sponges inhabiting Antarctic waters are still limited to a few species (but see Webster et al., 2004) and research on the microbiota of sub-Antarctic species is virtually nonexistent until now.

The aim of this study was to characterize and compare the microbiota of *Mycale* spp. (Mycalidae, Poecilosclerida, Demospongiae) inhabiting different geographic regions using targeted sequencing of the bacterial 16S rRNA gene. This was done using the Ion16S™ Metagenomics Kit (Thermo Fisher Scientific; Waltham, MA, USA) which allows the detection of a larger array of microbial groups, hence providing a more accurate description of the microbial community (Aloisio et al., 2016).

*Mycale* species are widely distributed at different latitudes (Hajdu, 1995; Riesgo et al., 2015), hence they constitute a valuable taxon for studies of the ecology and evolution of sponge symbiotic relationships, and how it is affected by environmental variation. Previous research on *Mycale* species from different latitudes, has characterized them as LMA species (e.g., Anderson, Northcote & Page, 2010; Erwin et al., 2015). Their capacity to host symbiotic microorganisms that produce bioactive compounds (e.g., Thomas, Kavlekar & LokaBharathi, 2010) or bioactive natural products with biotechnological potential (Habener, Hooper & Carroll, 2016) further enhances the importance of in depth studies on sponges of this genus.

The sponges *Mycale (Aegogropila) magellanica* (Ridley, 1881) and *Mycale (Oxymycale) acerata* Kirkpatrick, 1907 are both abundant members of shallow-water benthic communities at the southern tip of South America (Magellan region) and Antarctica (Cárdenas, 2012; Cárdenas et al., 2016; Cerrano et al., 2004; Dayton et al., 1974; Hajdu et al., 2016; Pansini & Sarà, 1999). Despite the information provided by Webster et al. (2004) on *M. acerata*, where a consistent pattern in the microbial community was reported at different sites in McMurdo Sound (East Antarctica), the bacterial communities associated with *Mycale* species from the WAP and the sub-Antarctic area of the Magellan region, remain largely unexplored. Considering that recent work has described host specificity (even in LMA sponges), here we assess if (1) the previously consistent pattern described for *M. acerata* from close locations in East Antarctica is maintained in the species from the WAP across a larger spatial scale (more than 370 km) and (2) if there is host specificity across other cold-water species from the genus *Mycale* from locations separated by more than 1,300 km.

## Materials and Methods

### Sample collection

**Figure 1:**
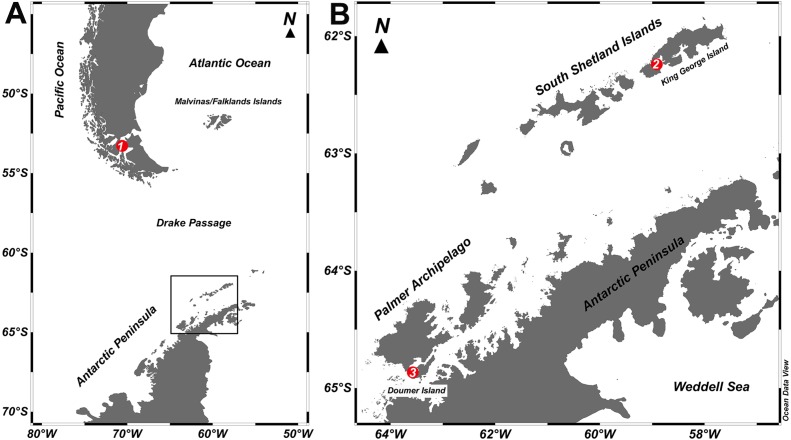
Study sites. Map of the locations where specimens of *Mycale (Aegogropila) magellanica* and *Mycale (Oxymycale) acerata* were collected in the (A) Magellan region and (B) the Western Antarctic Peninsula, respectively. 1, Rio Seco, Magellan Strait; 2, Fildes Bay, King George Island; 3, South Bay, Doumer Island.

Two specimens of *Mycale (Aegogropila) magellanica* were collected by SCUBA diving between 3 to 10 m depth at Rio Seco, Magellan Strait (53°03″38″S; 70°51″22″W) during the Austral summer 2015. Seawater temperature around the area ranges from 6 to 9 °C. Individuals of *Mycale (Oxymycale) acerata* were collected by SCUBA at 10 m depth at Fildes Bay (1 specimen), King George Island (62°12″17″S; 58° 56″47″W) and South Bay (1 specimen), Doumer Island, Palmer Archipelago (64°52″32″S; 63°35″02″W), Antarctica, also in the Austral summer 2015 (Fig. 1). Seawater temperature around the WAP ranged from −1.8 °C to 2 °C, although summer warming episodes have been reported for South Bay (Cárdenas, González-Aravena & Santibañez, 2018). At Magallanes, *Mycale* samples were collected from *Macrocystis pyrifera* beds at 5 m depth, whereas in Antarctica, samples were collected in *Himantothallus grandifolius* beds between 10 to 20 m depth. Samples were transported to the laboratory where pieces of sponge tissue (containing ectosome and choanosome layers) were immediately preserved in plastic tubes with RNAlater© (Sigma-Aldrich, St. Louis, MO, USA) for subsequent DNA extraction.

Sponge samples were transported to the laboratory at the Museu Nacional, Universidade Federal do Rio de Janeiro, where they were analysed and identified following the procedures detailed by Hajdu, Peixinho & Fernandez (2011).

The study was conducted under the permit 806/2015 granted by the Chilean Antarctic Institute (INACH).

### DNA extraction and sequencing

Genomic DNA from sponge tissue (≈0.5 g) was extracted using the Precellys© Evolution homogenizer (Bertin Technologies, Montigny-Le-Bretonneux, France) and a Power-Soil DNA Isolation Kit (MOBIO, Germantown, MD, USA), following the manufacturer’s instructions. DNA concentration was assessed using Qubit 2.0 fluorometer (Invitrogen, Carlsbad, CA, USA). Extractions were performed using both internal and external sponge tissue in order to obtain the whole bacterial community structure. The Ion 16S™ Metagenomics Kit was used, following the kit protocol for library preparation (Ion Torrent, 2014). The kit includes two primer sets amplifying the hypervariable regions V2, V3, V4, V6-7, V8 and V9 regions of the 16S rRNA gene. The high-throughput sequencing was performed by Uppsala Genome Centre (UGC), which is part of the Science for Life Laboratory (SciLifeLab, Uppsala, Sweden), using the IonTorrent PGM platform with an Ion 318™ Chip v2 and 400 bp read length chemistry. Sequences were deposited in NCBI SRA under the BioProject PRJNA397072.

### Sequence analysis

In order to avoid multiple sequences per putative OTU in the dataset, only one region (V3) containing the largest number of sequences was used for the majority of the analysis. However, a comparative view of taxonomic diversity of the different sequences is given in Table 1.

**Table 1:** Total reads and OTU richness. Number of total filtered reads and observed OTU richness over sequenced 16S regions from samples of *Mycale (Aegogropila) magellanica* and *Mycale (Oxymycale) acerata* from Rio Seco, Magallanes and from Fildes (King George Island) and South Bay (Doumer Island).

	Number of sequences								Number of OTUs							
	*M. acerata*				*M. magellanica*				*M. acerata*				*M. magellanica*			
Region	F1	Y1	Av.	St.dev.	MR2	MR4	Av.	St.dev.	F1	Y1	Av.	St.dev.	MR2	MR4	Av.	St.dev.
	867,074	1,354,520	1,110,797	344,676	815,387	781,153	798,270	24,207	3,332	3,932	3,632	424	3,694	4,310	4,002	436
V2	54,582	129,405	91,994	52,908	43,618	38,442	41,030	3,660	259	342	301	59	312	371	342	42
V3	327,785	678,025	502,905	247,657	342,045	330,286	336,166	8,315	1,867	2,314	2,091	316	2,107	2,506	2,307	282
V4	78,411	144,912	111,662	47,023	47,131	42,948	45,040	2,958	161	202	182	29	165	202	184	26
V6-7	134,094	104,409	119,252	20,990	146,808	110,190	128,499	25,893	587	634	611	33	608	711	660	73
V8	123,482	156,983	140,233	23,689	62,918	67,208	65,063	3,033	112	118	115	4	118	121	120	2
V9	143,644	120,103	131,874	16,646	141,416	175,517	158,467	24,113	322	300	311	16	361	363	362	1
Unassigned	5,076	20,683	12,880	11,036	31,451	16,562	24,007	10,528	24	22	23	1	23	36	30	9

Initial sequence quality was assessed using FastQC (Andrews, 2010). Data analysis of fastq sequence files was performed using the UPARSE pipeline with USEARCH 8.1.1861 (Edgar, 2013), including filtering (MaxEE = 0.5), minimum sequence length (180 bp), singleton and chimera removal and OTU clustering (97%). OTUs were sorted according to region, and chloroplast sequences were removed. Taxonomic assignment, alignment, V3 standardized depth resampling (290 K) and phylogenetic analyses were performed with QIIME (Caporaso et al., 2010), using the Silva v.128 database for taxonomical assignment.

### Statistical analysis

Univariate measures of diversity (Sobs, Chao1, Shannon and Simpson indexes) were calculated for each sponge to assess for differences between hosts. Similarity percentages analysis (SIMPER) was performed to identify OTUs contributing most to dissimilarity between samples of *M. magellanica* and *M. acerata*. Multidimensional scaling (MDS) plots were used to show variation in the composition of the microbiota of the studies species. In addition, permutational analysis of variance (PERMANOVA) (Anderson, 2001) based on Bray-Curtis distance matrix was performed to test for differences in the composition of the microbial communities among sponge species occurring in Antarctica and Magallanes. Statistical differences were tested using 9,999 permutations of raw data. For visualization and interpretation of the microbial community we used standardized 97% OTU abundance to plot a heatmap selecting the 20 most abundant OTUs. All analyses were performed using Primer v7 (Anderson, Gorley & Clarke, 2008).

## Results

### Seawater temperature

Seawater temperature at collection sites was remarkably different. Seawater temperature in Rio Seco, Magallanes was 8 °C, whereas seawater temperature at sites in Antarctica was 1.0 °C and 0.6 °C at Fildes and South Bay, respectively.

### Total reads and OTUs over all sequenced 16S regions

The mean total number of 97% OTUs distributed over all seven sequenced 16S regions was 4,002 ± 436 for *M. magellanica* and 3,632 ± 424 for *M. acerata.* The V3 region accounted for the majority of OTUs, with 2,091 ± 316 for *M. acerata* and 2307 ± 282 for *M. magellanica*, then the V6-7 region with 611 ± 33 and 660 ± 73 OTUs, the V9 region with 311 ± 15 and 362 ± 1 OTUs, the V2 region with 301 ± 59 and 342 ± 42 OTUs, the V4 region with 182 ± 29 and 184 ± 26 OTUs, and finally the V8 region with 115 ± 4 and 120 ± 2 OTUs (Table 1).

### V3 bacterial richness and diversity estimations

Richness estimates based on Chao1 was 2,396.6 ± 146.7 for *M. magellanica* and 2201.7 ± 67.1 for *M. acerata*. Rarefaction analyses suggested that the sequencing effort showed a high coverage for both species (Fig. S1).

Our analysis showed that the observed (Sobs) and estimated (Chao1) richness was slightly higher in *M. magellanica* (Magellan Strait) than in *M. acerata* (Antarctica) (Table 2). A similar situation occurred in terms of Shannon (H’) as values were 4.68 ± 0.79 and 4.49 ± 0.24 for *M. magellanica* and *M. acerata*, respectively. In contrast, Simpson (D) was very similar for both species, 0.969 ± 0.02 and 0.974 ± 0.0 for *M. magellanica* and *M. acerata*, respectively.

**Table 2:** Microbial diversity. Diversity values recorded in samples of *Mycale (Aegogropila) magellanica* and *Mycale (Oxymycale) acerata* from Rio Seco, Magallanes and from Fildes (King George Island) and South Bay (Doumer Island), WAP.

	*M. acerata*				*M. magellanica*			
	F1	Y1	Av.	St.dev.	MR2	MR4	Av.	St.dev.
Number of Seq	327,785	678,025	502,905	247,657	342,045	330,286	336,166	8,315
Number OTUs	1,867	2,314	2,091	316	2,107	2,506	2,307	282
Chao1	2,126	2,264	2,195	98	2,261	2,536	2,399	195
H’	4.324	4.664	4.494	0.240	4.123	5.245	4.684	0.793
D	0.970	0.974	0.972	0.003	0.954	0.984	0.969	0.021

**Notes.**

MR Rio Seco, Magellan Strait MA *Mycale (Oxymycale) acerata* F1 Fildes Y1 South Bay

### Composition of the total bacterial community associated with *Mycale* species

**Figure 2:**
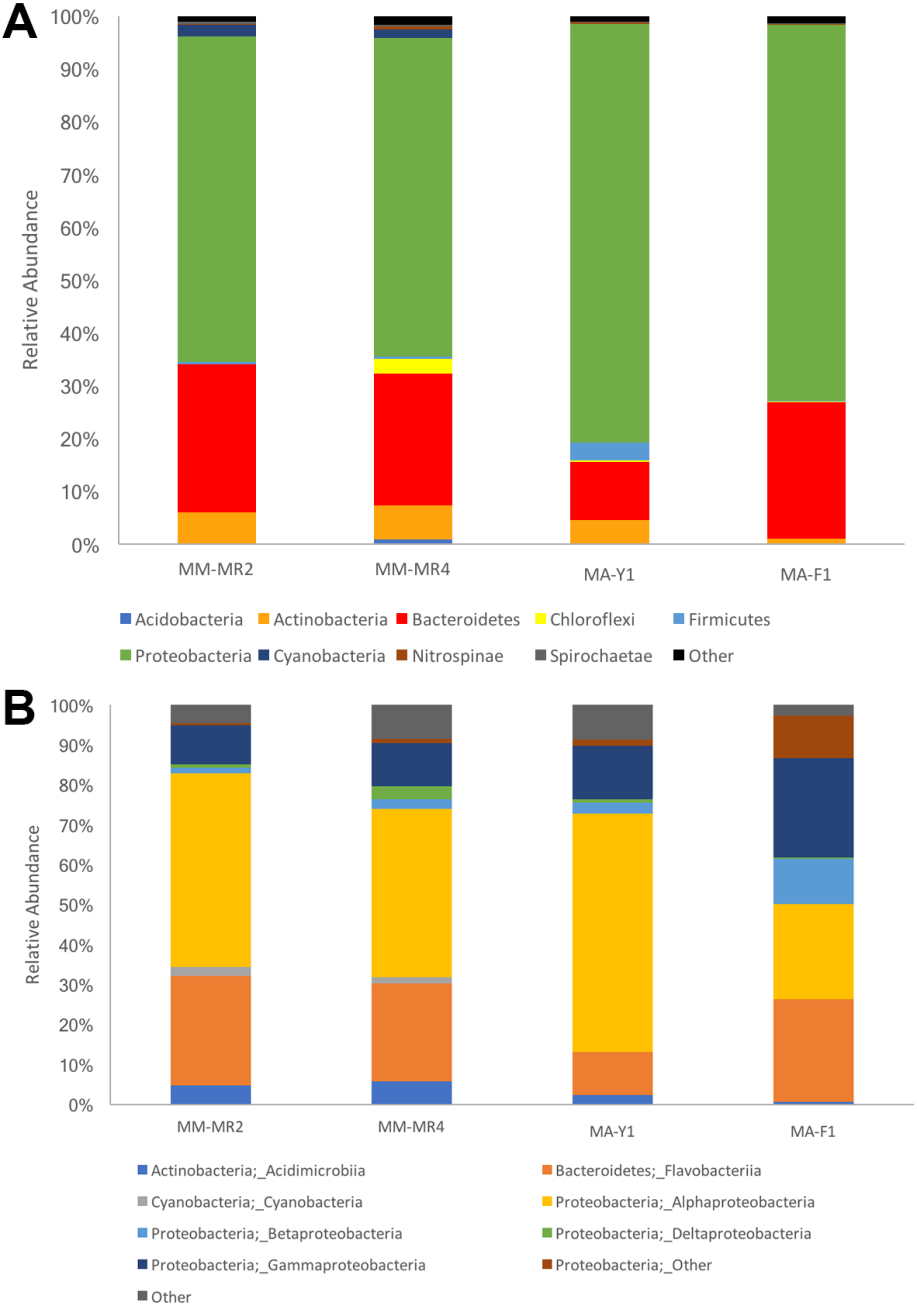
Microbial abundance. Relative abundance at (A) phylum and (B) class levels in samples of *Mycale (Aegogropila) magellanica* and *Mycale (Oxymycale) acerata* from Rio Seco, Magallanes and from Fildes (King George Island) and South Bay (Doumer Island), WAP. MM, *Mycale (Aegogropila) magellanica*; MR, Rio Seco, Magellan Strait; MA, *Mycale (Oxymycale) acerata*; F1, Fildes; Y1, South Bay.

The resolved OTUs were affiliated with bacterial sequences from 26 phyla, with *Proteobacteria* being the most dominant group reaching more than 69.1% of the abundance, followed by *Bacteroidetes* (22.4%), *Actinobacteria* (4.4%) and *Firmicutes* with the latter comprising 1.1% of the abundance (Fig. 2). In samples of the Antarctic sponge *M. acerata*, *Proteobacteria* ranged between 61.6% and 60.3%, whereas in the sub-Antarctic *M. magellanica* it ranged from 79.2% to 71.2%. Among this phylum, the most abundant classes were *Alpha-* and *Gammaproteobacteria* in both sponge species, with the former accounting for 59.6% to 23% of the abundance in *M. acerata*, whereas it ranged between 48.5% and 42.2% in *M. magellanica*. *Gammaproteobacteria* comprised 24.8% and 13.3% of sequences in samples of *M. acerata*, whereas its abundance ranged between 10.8% and 9.8% in *M. magellanica*. *Betaproteobacteria* was also abundant in *M. acerata* reaching more than up to 11% of the sequences, whereas in *M. magellanica* its abundance was lower*,* reaching between 1 to 2% of the abundance. The phylum *Bacteroidetes (Flavobacteria)* was also abundant, comprising between 25.8% and 11% of sequences in samples of *M. magellanica*, while it ranged between 27.9% and 25% in *M. acerata* (Fig. 2). *Acidimicrobiia (Actinobacteria)* were more abundant in *M. magellanica* than in *M. acerata*, while *Cyanobacteria* was only present in the former.

**Figure 3:**
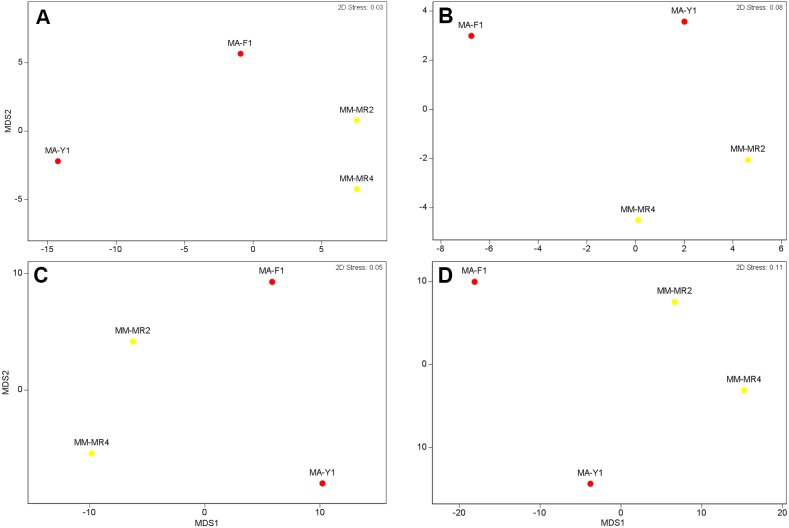
Microbial similarity. MDS plots showing similarity in microbial communities of *Mycale (Aegogropila) magellanica* and *Mycale (Oxymycale) acerata.* Presence absence data at (A) phylum and (B) class levels and relative abundance data at phylum (C) and class (D) levels. MM, *Mycale (Aegogropila) magellanica*; MR, Rio Seco, Magellan Strait; MA, *Mycale (Oxymycale) acerata*; F1, Fildes; Y1, South Bay.

Although samples from both species tended to separate in the ordination plots at the Phylum and Class levels, samples of *M. acerata* were not always grouped (Fig. 3). This explains the absence of a clear patter in our analyses as no significant differences between species either in terms of presence-absence (PERMANOVA_Phylum_  *F*_1,3_ = 3.4544, *P* = 0.3346, PERMANOVA_Class_  *F*_1,3_ = 1.9573, *P* = 0.2323) nor abundance were found (PERMANOVA_Phylum_  *F*_1,3_ = 2.1108, *P* = 0.2246, PERMANOVA_Class_  *F*_1,3_ = 1.4575, *P* = 0.3292).

**Figure 4:**
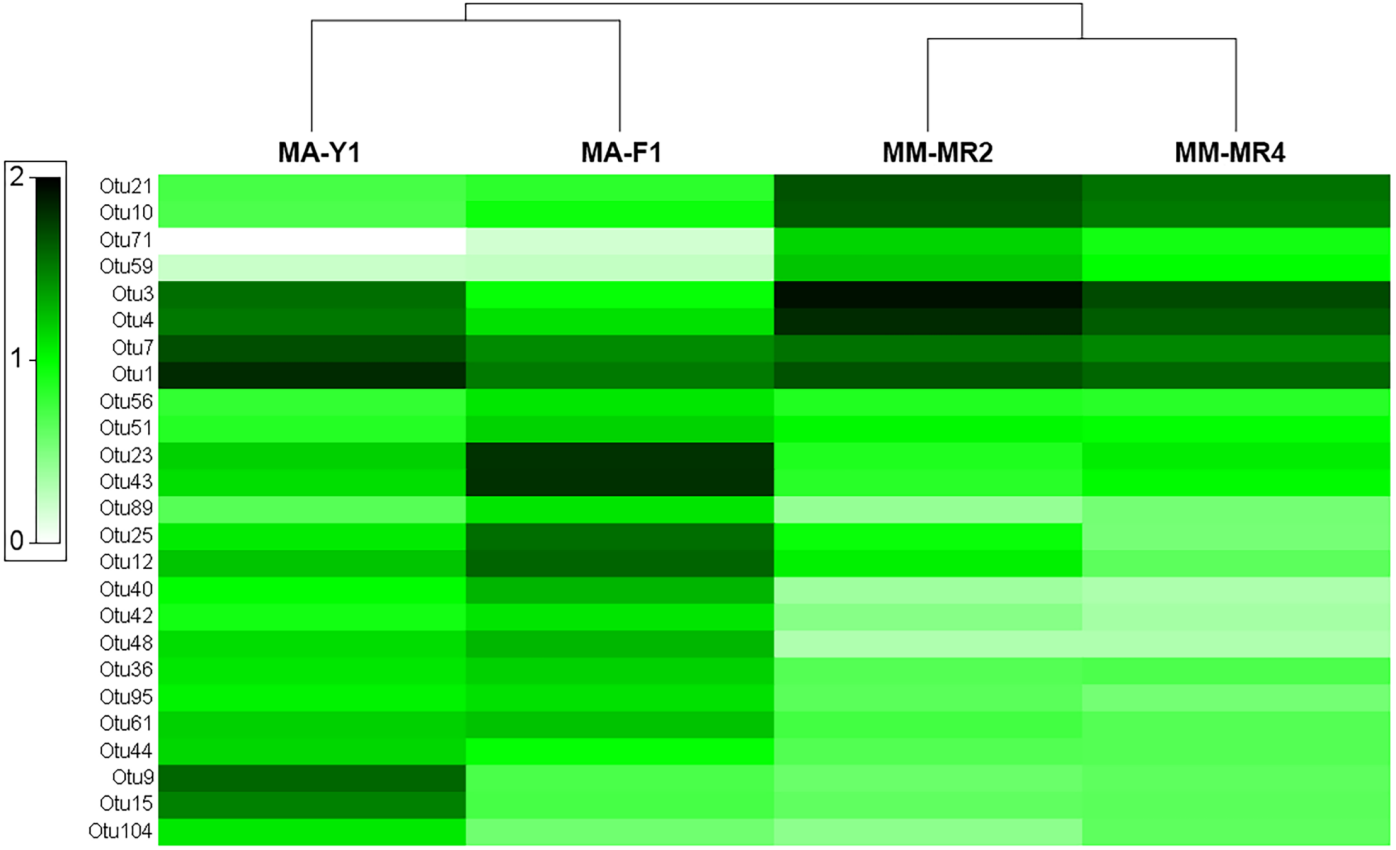
Microbial abundance. Heat map showing relative abundances of the 25 most abundant OTUs in samples of *Mycale (Oxymycale) acerata* and *Mycale (Aegogropila) magellanica* from Fildes (King George Island) and South Bay (Doumer Island), WAP, and Rio Seco, Magallanes. MM, *Mycale (Aegogropila) magellanica*; MR, Rio Seco, Magellan Strait; MA, *Mycale (Oxymycale) acerata*; F1, Fildes; Y1, South Bay. Color keys represent four root-transformed relative abundance (expressed as percentage). See Supplemental Information for detailed classification of OTUs.

**Figure 5:**
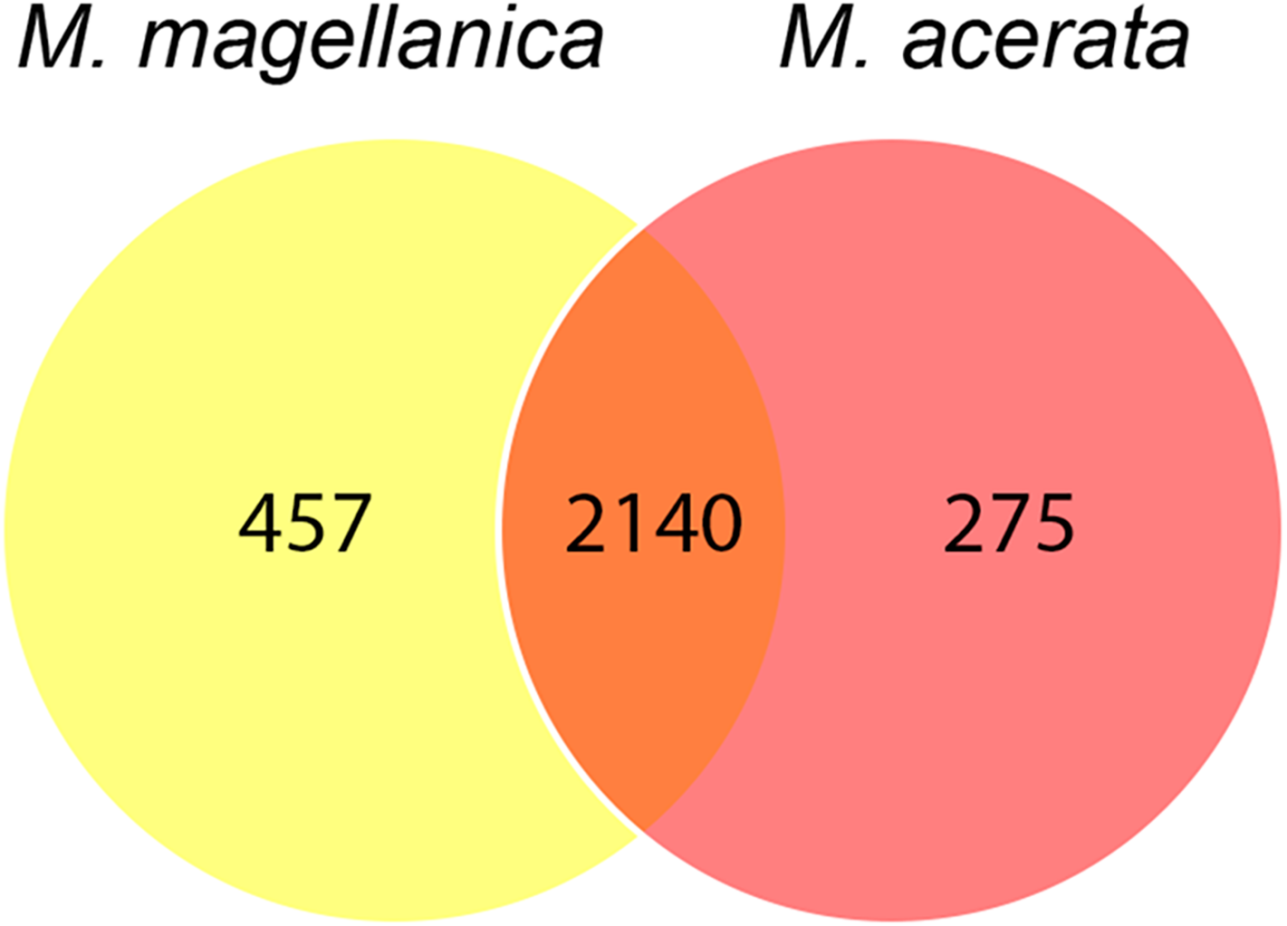
Venn diagram. Number of shared and unique OTUs recorded in samples of *Mycale (Aegogropila) magellanica* and *Mycale (Oxymycale) acerata* collected in the Magellan region and the Western Antarctic Peninsula, respectively.

The analysis at OTU level showed a similar situation, where some degree of variation in the abundance of OTUs was found when comparing samples, however this was not significant (Fig. 4, PERMANOVA *F*_1,3_ = 2.1593, *P* = 0.212). Similarly, no differences were found when data was transformed to presence-absence (PERMANOVA *F*_1,3_ = 2.27, *P* = 0.1975); Most OTUs, 74.7% of the total OTUs identified, were shared by both species. In contrast, 15.9% and 9.6% of the OTUs were only found in *M. magellanica* and *M. acerata*, respectively (Fig. 5). Interestingly none of the unique OTUs reached more than 1% of the abundance. These results demonstrate an important overlap among both *Mycale* species and also suggest the existence of a low level of specificity of most of the dominant symbiont groups.

The two most abundant OTUs in *M. magellanica* belonged to *Alphaproteobacteria* (*Rhodobacteraceae*) OTUs (OTU3 and OTU4), comprising approximately 11% and 9% of the total reads respectively. Another alphaproteobacterial OTU SAR Clade 11 (OTU1) and two *Flavobacteriaceae* (OTU21 and OTU10) were among the five most abundant. Similarly, OTU1 (SAR Clade 11) along with another alphaproteobacterial OTU (OTU7) were the two most dominant in *M. acerata,* accounting for 8 % and 6% of the abundance. Several of the most abundant OTUs, were also the most discriminant explaining the observed differences in the microbiota of both species (SIMPER Average dissimilarity = 62.23%, see Table 3 and Supplemental Information). For instance, OTU21 and OTU10 (*Flavobacteriaceae*) along with OTU3 (*Rhodobacteraceae*) were the three most discriminant OTUs, being responsible for 16% of the cumulative differences (Table 3). These three OTUs along with other 14 (mostly *Alpha- Gammaproteobacteria* and *Flavobacteria*), accounted for 50% of the cumulative differences between species. This shows a clear and dominant association of these bacterial groups within the microbial consortia living in *Mycale* sponges.

**Table 3:** Dominant OTUs. Seventeen most abundant bacterial OTUs in association with *Mycale (Aegogropila) magellanica* and *Mycale (Oxymycale) acerata* according to SIMPER. OTUs selected contributed to 50% of the dissimilarities between sponge species.

Taxa		MM	MA				
	OTU	Av.Abund	Av.Abund	Av.Diss	Diss/SD	Contrib%	Cum.%
Proteobacteria, Alphaproteobacteria, Rhodobacterales, Rhodobacteraceae	Otu3	11.13	3.42	3.85	1.79	6.19	6.19
Bacteroidetes, Flavobacteriia, Flavobacteriales, Flavobacteriaceae	Otu21	6.75	0.36	3.19	5.11	5.13	11.32
Bacteroidetes, Flavobacteriia, Flavobacteriales, Flavobacteriaceae; Ambiguous_taxa, Ambiguous_taxa	Otu10	6.35	0.51	2.92	4.47	4.69	16.02
Proteobacteria, Alphaproteobacteria, Rhodobacterales, Rhodobacteraceae	Otu4	9.12	3.44	2.84	1.76	4.57	20.58
Proteobacteria	Otu43	0.76	6.07	2.65	1.02	4.27	24.85
Proteobacteria, Betaproteobacteria, Nitrosomonadales, Nitrosomonadaceae, Candidatus Branchiomonas, uncultured marine bacterium	Otu23	0.93	6.11	2.59	1.06	4.16	29.01
Proteobacteria, Gammaproteobacteria, Enterobacteriales, Enterobacteriaceae, Buchnera	Otu12	0.69	4.41	1.86	1.44	3	32
Actinobacteria, Actinobacteria, PeM15, Ambiguous_taxa, Ambiguous_taxa, Ambiguous_taxa	Otu9	0.13	3.34	1.6	0.9	2.58	34.58
Proteobacteria, Gammaproteobacteria, Enterobacteriales, Enterobacteriaceae, Buchnera	Otu25	0.47	3.62	1.57	1.17	2.53	37.11
Proteobacteria, Alphaproteobacteria, SAR11 clade	Otu1	7.16	8.23	1.5	2.05	2.41	39.52
Proteobacteria, Alphaproteobacteria, Rhodobacterales, Rhodobacteraceae	Otu15	0.15	2.55	1.2	0.91	1.93	41.45
Bacteroidetes, Flavobacteriia, Flavobacteriales, Flavobacteriaceae, Polaribacter 1	Otu48	0.01	2.11	1.05	3.7	1.69	43.14
Proteobacteria, Alphaproteobacteria, SAR11 clade, Surface 1	Otu7	5.15	6.22	0.95	1.31	1.52	44.66
Proteobacteria, Gammaproteobacteria, Oceanospirillales, Oceanospirillaceae, Balneatrix, uncultured marine bacterium	Otu61	0.24	2.08	0.92	7.83	1.48	46.14
Bacteroidetes, Flavobacteriia, Flavobacteriales, Flavobacteriaceae, Polaribacter 1	Otu40	0.02	1.84	0.91	1.91	1.47	47.6
Actinobacteria, Acidimicrobiia, Acidimicrobiales, OM1 clade, Candidatus Actinomarina, uncultured bacterium	Otu59	1.59	0	0.8	2.17	1.28	48.88
Alphaproteobacteria, Rhizobiales, PS1 clade, Ambiguous_taxa, Ambiguous_taxa	Otu36	0.22	1.65	0.71	5.12	1.15	50.03

**Notes.**

MM *Mycale (Aegogropila) magellanica* MA *Mycale (Oxymycale) acerata*

## Discussion

The present study described for the first time the bacterial communities associated with two closely related cold-water sponges collected in Antarctica (62–64°S) and the sub-Antarctic area of South America (53°S). Although both *Mycale* species are rather conspicuous species in shallow-water habitats around Antarctica and also in Magallanes, and hence are expected to play important roles in these habitats (see Bell, 2008), the microbiota associated with these species have not been well studied. The only previous information available on *M. acerata* from East Antarctica was provided by Webster et al. (2004) based on clone libraries and denaturing gradient gel electrophoresis (DGGE) analyses. In addition, the taxonomic richness (OTUs) presently recorded from *M. acerata* and *M. magellanica* is similar to what was reported for other eight Antarctic sponges (Rodríguez-Marconi et al., 2015), confirming that cold-water sponges can host diverse and microbial communities. These authors used high throughput sequencing to describe the microbial community of specimens from Fildes Bay (King George Island, Maritime Antarctic), none of which belonging to *Mycale*. Although our study did not analyze the microbial communities from seawater, we could expect the existence of a similar pattern as those described for other Antarctic sponges (see Rodríguez-Marconi et al., 2015; Webster et al., 2004). Webster et al. (2004) described a high proportion of banding patterns in *M. acerata* that were not found in the surrounding seawater. This needs further confirmation especially for the sub-Antarctic sponge *M. magellanica*, as no information about seawater communities is available for the Magellan Strait. Such analysis could be important considering some LMA sponges host less specific microbiota (more similar to those found the in surrounding seawater) (Giles et al., 2013), which also tend to be more flexible over time (Erwin et al., 2015).

The use of the Ion Torrent platform enabled us to compare data obtained from seven different 16S regions showing variable results. The V3 region accounted for the majority of OTUs with over 2000 OTUs, whereas regions V4 and V8 accounted for less than 200 OTUs. This shows the importance of the region selected for the analysis and how results and interpretation will be affected depending on this. The use of multiple variable regions provides complete information allowing the detection of more diverse bacterial communities and preserves the information from single V-regions for a multi-dimensional analysis (Aloisio et al., 2016; Barb et al., 2016). In this study, we focused on V3 because it was the most diverse, which is important considering this is the first time the microbiota of both species is reported.

### Composition of the microbiota of *Mycale* species compared with other sponges

In general, our results are in accordance with other Antarctic sponge species collected around Fildes Bay, King George Island (Rodríguez-Marconi et al., 2015), where *Proteobacteria* is highly dominant in terms of abundance. In this regard, based on the abundance of dominant groups, both *Mycale* species could be classified as LMA sponges, which are commonly characterized by the presence of communities dominated by a few groups (e.g., *Proteobacteria, Bacteroidetes*) (Cárdenas et al., 2014; De Mares et al., 2017; Erwin et al., 2012b; Giles et al., 2013; Moitinho-Silva et al., 2014; Moitinho-Silva et al., 2017; Rodríguez-Marconi et al., 2015; Sipkema et al., 2009); however, further analysis may provide more information about the density of these groups and also will help to confirm the status of these species. Our results are in accordance with the status of LMA for *Mycale* species from other latitudes (e.g., Anderson, Northcote & Page, 2010; Erwin et al., 2015; Moitinho-Silva et al., 2017). This status may be supported by the fact that *Poribacteria* which is normally associated with HMA sponges (Moitinho-Silva et al., 2014; Moitinho-Silva et al., 2017), was not found in our samples. However, based on microbiota analyzed from several other sponge species, it seems that this group is absent (or at least very rare) in Antarctic environments, since it has not been found in other Antarctic sponge species studied so far (Rodríguez-Marconi et al., 2015).

A recent study on *Mycale* sp. from the southeast coast of South Africa revealed that a single betaproteobacterial OTU dominated the bacterial community of this sponge, accounting for up to 84% of the sequence reads obtained (Matcher et al., 2017). A similar observation has been reported in other species such as the well-studied Mediterranean sponge *Crambe crambe* (Croué et al., 2013) and the Antarctic *Latrunculia apicalis* (Webster et al., 2004). Sponges belonging to Mycalidae and other families within the order Poecilosclerida (including Crambeidae and Microcionidae), also harbor bacterial communities dominated by betaproteobacterial symbionts (Matcher et al., 2017). In contrast, the pattern observed in this study is slightly different, where microbial communities are dominated by a few OTUs rather than a single one, with alphaproteobacterial OTUs as the most dominant OTUs in *M. acerata* and *M. magellanica*. While the microbiota of *M. magellanica* was dominated by alphaproteobacterial (Rhodobacteraceae and SAR11 Clade) and flavobacterial OTUs, the most abundant OTUs in *M. acerata* were alpha-, gamma- and betaproteobacterial OTUs. This is similar to what has been reported for other species such as *Mycale hentscheli* from New Zealand, which is also dominated by *Alpha-* and *Gammaproteobacteria* (Anderson, Northcote & Page, 2010).

Alphaproteobacterial OTUs belonging to *Rhodobacteraceae* were abundant in both species, especially in *M. magellanica*. The relative abundance of the two most dominant OTUs (OTU3 and 4) in M. *magellanica* comprised 11% and 9% of the total abundance respectively, which is similar to the values reported for other Antarctic sponges such as *Hymeniacidon torquata* (11%), *Haliclona (Gellius)* sp. (14%) from Fildes Bay, King George Island (Rodríguez-Marconi et al., 2015). The same two OTUs along with two other *Rhodobacteraceae* (OTU 9 and 15) were among the 10 most dominant OTUs in *M. acerata.* It is known that marine *Rhodobacteraceae* are key players in biogeochemical cycling and are often mutualist with eukaryotes (Simon et al., 2017), however no further information is available on these specific OTUs to confirm this situation.

### Similarity in the microbiota of *M. magellanica and M. acerata*

Several studies have addressed spatial and temporal dynamics in the microbiota of different species, including comparisons between HMA and LMA species (Erwin, Olson & Thacker, 2012; Marino et al., 2017; Montalvo & Hill, 2011; Souza et al., 2017), and also addressing the host specificity in phylogenetically closely related sponges (Easson & Thacker, 2014; Thomas et al., 2016). Although, generally HMA tend to show a higher degree of host specificity than LMA sponges (see Björk et al., 2013; Giles et al., 2013; Erwin et al., 2015), some research has reported similar host specificity for species belonging to both categories (De Mares et al., 2017; Easson & Thacker, 2014). For instance, Marino et al. (2017), studying the HMA sponge *Ircinia campana* from contrasting environments (from temperate to tropical Caribbean habitats) found a consistent significant pattern of dissimilarity with distant habitats, suggesting differences in environmental conditions may exert different selective pressures on sponge holobionts. In our study, although some degree of variability was recorded between the samples of *M. acerata* from King George Island and the Palmer Archipelago, the microbiota of the samples from both localities was similar. Webster et al. (2004) using different molecular techniques than those currently employed, reported consistent patterns in the microbial communities of *M. acerata* from three sites around East Antarctica. In contrast, studies of *Mycale hentscheli* from New Zealand (Anderson, Northcote & Page, 2010) reported clear differences between localities, suggesting that observed spatial variability in the bacterial communities of *M. hentscheli* may be influenced by differences in seawater temperature in the studied sites that ranged from subtropical (20 °C–14 °C) to cold-temperate waters in New Zealand (15 °C–8 °C). Interestingly, it seems this does not apply to *M. acerata*, and perhaps to other cold-water sponges. In this regard, further spatial and temporal studies should characterize core and variable microbial components of the symbiont communities of these species (see Thomas et al., 2016; Astudillo-García et al., 2017), which are more tractable over temporal scales (Erwin et al., 2015), to provide a better understanding of the dynamics of the microbiota of both *Mycale* species.

Different studies have provided evidence that closely related sponges tend to host less dissimilar microbial communities (Erwin, Olson & Thacker, 2012; Montalvo & Hill, 2011; Souza et al., 2017). Montalvo & Hill (2011) reported a significant similarity in sponge-associated bacteria from two *Xestospongia* species from Indonesia and Florida (USA). The referenced study reported that 26% of OTUs were shared by the two *Xestospongia* species, which are considered HMA sponges. Our results show an even higher degree of similarity between both *Mycale* species (sharing more than 74% of the OTUs), which is remarkable considering both species occur in geographically distant areas (>1,300 km), with significant environmental differences in terms of seawater temperature. In general, despite slight differences in the abundance, the most dominant OTUs were present in both species, whereas the unique OTUs had very low abundances. The high similarity in the microbiota of both species might be surprising considering these species have evolved in two distant regions with considerably dissimilar environmental conditions. However, despite both regions being isolated and shaped by geological and climatic atmospheric factors, there is evidence of close biogeographic links (e.g., geological, biogeographic) between the marine fauna of Antarctica and the Magellan region (see Arntz, Lovrich & Thatje, 2005; Downey et al., 2012). We could speculate that sponge-microbial communities may have evolved with their host in these cold-water environments (and highly stable in the case of the Antarctic), however further research is needed as host-microbiome systems seem to be influenced by highly complex interactions and effects which are not fully understood (Douglas & Werren, 2016). In addition, a wider coverage of sites, more replicates and seawater samples will help to improve our understanding on how the microbial communities of closely related sponge species are shaped in terms of similarities and dissimilarities. Given that *M. magellanica* is found in both areas considered in the present study, it would be important to compare samples of this species in both areas to confirm the importance of host and the connection between both geographic areas. This information will be very important to provide new insights on the relationship between host identity and evolutionary history of the host in cold-water sponges, especially considering recent evidence (see Easson & Thacker, 2014; Thomas et al., 2016), mainly based on tropical and temperate sponges, suggest that although phylogenetic relationships play an important role in structuring microbial diversity, host identity seems to be critical since specific factors also seem to influence the associated microbial community.

## Conclusions

The presence of highly similar microbiota in species collected from distant areas with contrasting environmental conditions and the LMA status of these species, which are often less complex and hence more tractable (Erwin et al., 2015), makes them interesting models to study how species such as *M. acerata* which have evolved in a very cold and highly stable environment and how they might respond to changes in environmental conditions. In this regard, it is important to develop further experiments that may provide additional insights on host specificity and the potential resilience of cold-water sponge species to environmental stress produced by projected climate change scenarios.

##  Supplemental Information

10.7717/peerj.4935/supp-1 Figure S1 Microbial richness Observed (A) and estimated (B) microbial richness in samples of Mycale (Aegogropila) magellanica (blue) and Mycale (Oxymycale) acerata (red) from Rio Seco, Magallanes and from Fildes (King George Island) and South Bay (Doumer Island), WAP.

10.7717/peerj.4935/supp-2 Table S1 Microbial abundance List of the 25 most abundant OTUs in samples of *Mycale (Oxymycale) acerata* and *Mycale (Aegogropila) magellanica* as shown in Fig. 4.

10.7717/peerj.4935/supp-3 File S1 OTUs in *Mycale* spp Relative abundance and taxonomy of 97% OTUs in samples of *Mycale (Oxymycale) acerata* and *Mycale (Aegogropila) magellanica*.

## Additional Information and Declarations

### Field Study Permissions

The following information was supplied relating to field study approvals (i.e., approving body and any reference numbers): The study was conducted under the permit 806/2015 granted by the Chilean Antarctic Institute (INACH).

### DNA Deposition

The following information was supplied regarding the deposition of DNA sequences: Sequences were deposited in NCBISRA under the BioProject PRJNA397072.

### Data Availability

The following information was supplied regarding data availability: The raw data are provided in a Supplemental File.
