# Supplementary figures and images for: High similarity in the microbiota of cold-water sponges of the Genus Mycale from two different geographical areas
Source: PeerJ. 2018 Jun 7;6:e4935. doi: 10.7717/peerj.4935 (PMC5994334; doi:10.7717/peerj.4935)

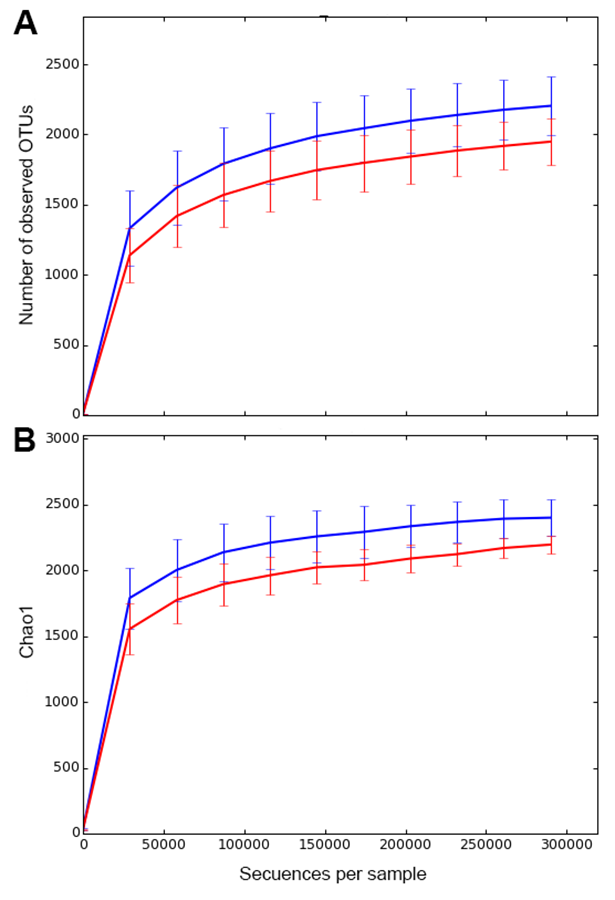

Supplement: Figure S1 — Observed (A) and estimated (B) microbial richness in samples of Mycale (Aegogropila) magellanica (blue) and Mycale (Oxymycale) acerata (red) from Rio Seco, Magallanes and from Fildes (King George Island) and South Bay (Doumer Island), WAP. [file peerj-06-4935-s001.png]
