# Supplementary material for: High similarity in the microbiota of cold-water sponges of the Genus Mycale from two different geographical areas
Source: PeerJ. 2018 Jun 7;6:e4935. doi: 10.7717/peerj.4935 (PMC5994334; doi:10.7717/peerj.4935)
Supplement: Table S1 — List of the 25 most abundant OTUs in samples of Mycale (Oxymycale) acerata and Mycale (Aegogropila) magellanica as shown in Fig. 4. [file peerj-06-4935-s002.docx]

| #OTU ID | Taxa |
| --- | --- |
| 21 | Bacteroidetes;_Flavobacteriia;_Flavobacteriales;_Flavobacteriaceae |
| 10 | Bacteroidetes;_Flavobacteriia;_Flavobacteriales;_Flavobacteriaceae; Ambiguous_taxa; Ambiguous_taxa |
| 71 | Bacteroidetes;_Bacteroidetes VC2.1 Bac22;Other;Other;Other |
| 59 | Actinobacteria;_Acidimicrobiia;_Acidimicrobiales;_OM1 clade;_Candidatus Actinomarina;_uncultured bacterium |
| 3 | Proteobacteria;_Alphaproteobacteria;_Rhodobacterales;_Rhodobacteraceae |
| 4 | Proteobacteria;_Alphaproteobacteria;_Rhodobacterales;_Rhodobacteraceae |
| 7 | Proteobacteria;_Alphaproteobacteria;_SAR11 clade;_Surface 1 |
| 1 | Proteobacteria;_Alphaproteobacteria;_SAR11 clade |
| 56 | Actinobacteria;_Actinobacteria;_Propionibacteriales;_Nocardioidaceae;_Kribbella |
| 51 | Actinobacteria;_Actinobacteria;_Micrococcales;_Micrococcaceae;_Pseudarthrobacter |
| 23 | Proteobacteria;_Betaproteobacteria;_Nitrosomonadales;_Nitrosomonadaceae;_Candidatus Branchiomonas;_uncultured marine bacterium |
| 43 | Proteobacteria |
| 89 | Bacteroidetes;_Bacteroidia;_Bacteroidales;_Rikenellaceae |
| 25 | Proteobacteria;_Gammaproteobacteria;_Enterobacteriales;_Enterobacteriaceae;_Buchnera |
| 12 | Proteobacteria;_Gammaproteobacteria;_Enterobacteriales;_Enterobacteriaceae;_Buchnera |
| 40 | Bacteroidetes;_Flavobacteriia;_Flavobacteriales;_Flavobacteriaceae;_Polaribacter 1 |
| 42 | Actinobacteria;_Actinobacteria;_Micrococcales;_Intrasporangiaceae |
| 48 | Bacteroidetes;_Flavobacteriia;_Flavobacteriales;_Flavobacteriaceae;_Polaribacter 1 |
| 36 | Alphaproteobacteria;_Rhizobiales;_PS1 clade; Ambiguous_taxa; Ambiguous_taxa |
| 95 | Bacteroidetes;_Cytophagia;_Cytophagales;_Flammeovirgaceae;_Marinoscillum |
| 61 | Proteobacteria;_Gammaproteobacteria;_Oceanospirillales;_Oceanospirillaceae;_Balneatrix;_uncultured marine bacterium |
| 44 | Actinobacteria;_Actinobacteria;_Micrococcales;_Intrasporangiaceae;Other |
| 9 | Actinobacteria;_Actinobacteria;_PeM15; Ambiguous_taxa; Ambiguous_taxa; Ambiguous_taxa |
| 15 | Acidobacteria;_Subgroup 26;_uncultured bacterium;_uncultured bacterium;_uncultured bacterium |
| 104 | Bacteroidetes;_Flavobacteriia;_Flavobacteriales;_Cryomorphaceae;_Owenweeksia |
